# Interaction between temperature and sublethal infection with the amphibian chytrid fungus impacts a susceptible frog species

**DOI:** 10.1038/s41598-018-35874-7

**Published:** 2019-01-14

**Authors:** Lachlan Campbell, Deborah S. Bower, Simon Clulow, Michelle Stockwell, John Clulow, Michael Mahony

**Affiliations:** 1 School of Environmental and Life Sciences, University of Newcastle, Callaghan, Newcastle, 2300 NSW Australia; 2 James Cook University, Townsville, 4811 Qld Australia; 3 Department of Biological Sciences, Macquarie University, Sydney, NSW 2109 Australia

## Abstract

The amphibian chytrid fungus *Batrachochytrium dendrobatidis* is an emerging infectious pathogen present on every continent except Antarctica. It causes the disease chytridiomycosis in a subset of species but does not always result in disease or death for every host. Ambient temperature influences both amphibian metabolism and chytrid pathogenicity, however the interactive effects on host physiology is not well understood. We investigated the sublethal effect of *B*. *dendrobatidis* infection on a susceptible host, *Litoria aurea* to test (1) whether the infection load, metabolic activity, body fat and gonad size differed in *L*. *aurea* at either 24 °C or 12 °C ambient temperatures and (2) whether previous *Bd* infection caused long-term changes to body fat and gonad size. *Litoria aurea* in 12 °C treatments had higher infection loads of *B*. *dendrobatidis* and lower survivorship. Metabolic rate was higher and fat mass was lower in infected individuals and in animals in 24 °C treatments. Male *L*. *aurea* previously infected with *B*. *dendrobatidis* had smaller testes 5 months-post clearance of infection, an effect likely to translate to fitness costs in wild populations. These experiments demonstrate a physiological cost to sublethal *B*. *dendrobatidis* infection, which suggests a reduction in host fitness mediated by temperature in the host’s environment regardless of whether infection leads to mortality.

## Introduction

Chronic sublethal infections can cause a range of deleterious impacts in the host^1^, placing a substantial and often unpredictable stress on host fitness^2^. Such stress can result in the redistribution of energy away from reproduction, investing instead in high priority resources such as immunity and the nervous system^3,4^, ultimately affecting host population dynamics^5^. Persistent and sublethal infections may dramatically alter the behaviour and physiology of host species causing both direct reductions in survival, or affecting fitness through reductions in reproductive potential^6^. Hosts also exhibit costly defence responses against parasites, including complex immune systems, behavioural and chemosensory driven avoidance, fever responses and terminal investment strategies to increase fitness^7^. These complex interactions in species susceptible to emerging infectious diseases will be better understood by first understanding the key driving mechanisms in which pathogens directly affect their host.

The amphibian chytrid fungus *B*. *dendrobatidis* infects a multitude of amphibian hosts around the world^8^ and has been detected in over 42% of the 1240 species currently surveyed^9^. It causes the disease chytridiomycosis in a subset of species susceptible to increases in infection loads beyond a tolerance threshold^10–12^. However, infection does not necessarily result in disease or death in all individuals, including susceptible species^13^, and in some individuals of such species the epidermal load of *B*. *dendrobatidis* can fluctuate without resulting in obvious symptoms or harm^10,14^. Progression of infection is influenced by the external environment, and ambient temperature can strongly influence the infection load of ectothermic hosts^15–17^. While *B*. *dendrobatidis* grows optimally at 17–25 °C^18^ in culture media, interactions between frog immune systems, the skin microbiome^19^ and pathogen growth at different temperatures can result in higher infection loads at lower temperatures (below pathogen thermal optima) where host-immunity is inhibited^20–22^, or in slower growth in the optimal pathogen temperature range when frog immune function is greater, even allowing susceptible individuals to sometimes clear infections^23,24^. Host responses to infection must therefore be considered within the context of the environment, particularly ambient temperature, given its ability to drive pathogen-specific traits as well as ectothermic host responses^25^.

Whilst *B*. *dendrobatidis* has the potential to induce sublethal responses, our understanding of the effects of infection on the physiology of individuals is poor. Previous studies on sublethal impacts of *Batrachochytrium dendrobatidis* infection have demonstrated lower respiration rate^12^, reduced body mass^26,27^ and locomotion metrics^28^ and retarded growth rates^29,30^. Reproductive metrics such as calling activity may increase in infected individuals^31^ while call frequency remains unchanged^32^. Spermatogenesis and oogenesis can increase in diseased individuals suggesting a terminal investment strategy^33^, while in other cases there are no spermatogenic differences between infected and uninfected individuals^28^. While much research has focused on *B*. *dendrobatidis* as a direct driver of population declines through increased mortality, understanding such sublethal effects of infection is crucial to understanding how the fitness of populations of susceptible species that persist with the disease and even relatively unaffected reservoir species might be challenged. Given the influence of temperature on host – parasite interactions^25^ and the energetically costly effects of sublethal infections^3,4^, it is important to understand the metabolic responses of *B*. *dendrobatidis* infection in the context of the ambient environmental temperature of the host.

The objective of this study was to explain the causes of previously demonstrated sublethal effects of *B*. *dendrobatidis* infection by focussing on metabolic and temperature interactions of a susceptible species^34,35^, the green and golden bell frog, *Litoria aurea*. Specifically, we aimed to test (1) whether the infection load, metabolic activity, and fat storage differed between *L*. *aurea* at high (24 °C) and low (12 °C) ambient temperatures following infection with *B*. *dendrobatidis* and (2) whether body fat and gonad size differed in frogs previously infected, but subsequently cleared of infection.

## Methods

### Experiment 1: Sublethal *B*. *dendrobatidis* infection of sexually immature *L*. *aurea* at winter (12 °C) and summer (24 °C) mean temperatures

#### Husbandry and experimental setup

*Litoria aurea* were bred from second generation captive breeding stock (derived from Kooragang Island, New South Wales, Australia, latitude: −32.862911°S, longitude: 151.728685°N) and reared at the University of Newcastle, New South Wales. Offspring were housed in groups of approximately 200 tadpoles in 450 × 300 × 150 mm aquaria as tadpoles under laboratory conditions with a 12 hour light/dark regime and were fed a mixed diet of boiled lettuce and trout pellets *ad libitum*. All individuals were transferred to 170 × 120 × 75 mm aquaria maintained under laboratory conditions post-metamorphosis. Eighty frogs that were 6–10 weeks post-metamorphosis were selected at random from 8 different clutches and placed individually into separate transparent plastic aquaria (170 × 120 × 75 mm). Aquaria contained autoclaved deionised water and pebbles as the substrate, and were tilted so that water covered 50% of the base in each aquarium. Each individual was allocated randomly to avoid effects of clutch to one of the four experimental groups (n = 20 per treatment) according to a two-way factorial design with temperature (12 °C or 24 °C) and *B*. *dendrobatidis* exposure (infected or uninfected) as treatments. The temperature treatments were selected because they are the approximate means of winter and summer in the sourced frogs’ range respectively. Each aquarium was maintained in an illuminated, refrigerated incubator (TRISL – 1175, Thermoline Scientific Equipment, Wetherill Park, NSW) with air-temperature set to 12 °C or 24 °C. The incubator cycled on a 12 hour light/dark regime and the temperature was monitored continuously within each incubator. Each day, all aquaria were randomly reallocated to a new position in the temperature cabinet in order to normalise any differences in temperature and lighting within the cabinet. Aquarium water was changed every 2–3 days and frogs were provided with 4–6 small, calcium powder dusted crickets twice per week. The condition and health of each individual was monitored daily.

#### Batrachochytrium dendrobatidis cultivation and inoculation

Samples of *B*. *dendrobatidis* (strain: Gibbo River-Llesueuri-00-LB-1, passage number 4) were obtained and cultured on TGhL agar plates, which were then flooded with sterilised water to harvest the actively growing zoospores. Exposure treatment individuals (n = 40) received a single inoculate with *B*. *dendrobatidis* by aliquoting 2 mL of the zoospore suspension directly into the aquarium water of each treatment individual. A 100 µl sample of the *B*. *dendrobatidis* suspension stock solution was used to quantify the concentration of zoospores using a haemocytometer, which was determined to be 9.05 × 10^6^ cells/mL. For sham controls, sterile TGhL agar plates were prepared and maintained under the same conditions as the plates containing active *B*. *dendrobatidis*. The sterile plates were flooded with sterilised water and 2 mL of the sham solution was aliquoted into the water bath of each control individual as a sham inoculation. Water changes were postponed for approximately 10 days after addition of *B*. *dendrobatidis* or sham inoculations. All individuals were closely monitored after inoculation for signs of rapid onset of disease.

#### Batrachochytrium dendrobatidis detection and quantification

All individuals were tested for infection prior to inoculation with either the *B*. *dendrobatidis* or sham solutions to ensure that individuals were naïve to their corresponding treatments. A standardised swabbing technique was employed over the epidermal surfaces prone to high infection loads, which involved wiping both sides of the ventral skin 8 times, the inner and outer thighs 4 times and the hind and fore feet 2 times each^10^. DNA extraction and quantification from the swabbed samples was conducted following standard qPCR Taqman assay methods^36^, using a Rotor Gene 6000 Real Time DNA amplification system. Each swab was assessed for the presence of *B*. *dendrobatidis* in triplicate using a 1/10 diluted sample from the extracted DNA, and are given as the mean chytrid DNA molecules per microlitre (molecules/µl) of a standardised extract volume (5 µl) from three replicates of the same swab sample. To generate standard curves, calibration standards were obtained from Pisces Molecular (Boulder, CO, USA) and were serially diluted to prepare four known *B*. *dendrobatidis* concentrations (18700, 1870, 187 and 18.7 molecules/microliter). Where replication of *B*. *dendrobatidis* DNA occurred in all three replicates, the number of molecules per microliter was summarised as the mean infection load. Where a result occurred with amplification in two of three replicates and the negative template control revealed no contamination, the sample was considered positive and the mean was calculated from all three replicates. Results with amplification of only one replicate were considered negative for *B*. *dendrobatidis* DNA. It was necessary to include zero values in the calculations as it was assumed to be the result of a low quantity of DNA in the sample^10,37^. All values were multiplied by 10 to account for the dilution step. Negative results were recorded for samples if all three replicates did not amplify, providing that the sample was not inhibited. To test for inhibition, internal positive controls were included in one of the replicates for each sample including the negative template control. Following qPCR, a threshold was set midway up the amplification curve, where inhibition was confirmed if the sample crossed the threshold more than five cycles after the negative template control. If the sample was inhibited, a 1/100 dilution with sterile Milli-Q water was performed on the originally extracted sample to dilute any potential inhibiting compounds and the qPCR process was repeated. The result of the diluted sample was multiplied by 100 to factor for the dilution step.

#### Batrachochytrium dendrobatidis infection loads and survival

Each frog was swabbed and tested for infection loads at 7, 17, 39 and 48 days post-exposure to the *B*. *dendrobatidis* or sham inoculations. Survival at 48 days was recorded.

#### Standard metabolic rate

Eight individuals were randomly selected from each of the four experimental treatment groups forty-one days post-exposure to *B*. *dendrobatidis* to measure the standard metabolic rate (SMR), however only the surviving 8 individuals were used for the 12 °C infected group. Individual frogs were fasted for six days before standard metabolic rate measurements to ensure they were in a post-absorptive state^38^. Each individual was placed in a 100 ml Schott bottle chamber that contained 80 ml of agarose gel, added for the purpose of reducing excess volume of the chamber to improve the accuracy of the reading. The chamber was sterilised between individuals by boiling the agarose gel in a microwave to prevent any cross-contamination of *B*. *dendrobatidis*. Each chamber also contained filter paper with 1 ml of deionised water to prevent desiccation of frogs during the procedure. Individual metabolic chambers containing frogs were immersed in a water bath at either 12 °C or 24 °C according to their allocated experimental temperature treatment. The rate of oxygen consumption in each metabolic chamber was measured by attaching the chamber to a computer-controlled, closed-circuit, indirect respirometer (Micro Oxymax, Columbus Instruments, Columbus, OH). The respirometer was manually calibrated each time the experiment was repeated. All metabolic measurements were conducted during daylight hours (09:00–19:00 h) to maintain consistency of readings within the diurnal phase of the diel cycle for each treatment group. The oxygen consumption of each individual was monitored for five hours, but only data from the last four hours were recorded. After the final data point was recorded each individual was weighed and returned to its aquarium. The SMR was calculated by dividing the rate of oxygen consumption (O_2_ ml/h) by the body mass of the individual (g) raised to the power of 0.75^39^.

1 $${\rm{Standard}}\,{\rm{Metabolic}}\,{\rm{Rate}}=\frac{{{\rm{O}}}_{2}{\rm{ml}}/{\rm{h}}}{{({Mass})}^{0.75}}$$

#### Euthanasia and determination of abdominal fat mass

After 48 d post-exposure to the *B*. *dendrobatidis* or sham inoculation, all surviving frogs were euthanized via immersion in tricaine methanesulfonate (MS-222; Sigma-Aldrich). The fat bodies were surgically removed under a dissecting microscope, blot-dried and weighed whole on an analytical balance ( ± 0.0001 g).

### Experiment 2: Long-term sublethal effects of a previous *B*. *dendrobatidis* infection in *L*. *aurea*

*Litoria aurea* (n = 84) were investigated to examine the chronic effect of a previous *B*. *dendrobatidis* infection on gonad mass. Frogs were obtained from the same captive colony described in experiment 1 and also followed the same animal husbandry, *B*. *dendrobatidis* cultivation, inoculation, detection and quantification procedures. There was no experimental temperature treatment for experiment 2 and all frogs were held at 20 °C. *Batrachochytrium dendrobatidis* exposed (n = 48) and sham exposed (n = 48) frogs were maintained for 95 days prior to undergoing a standard heat-treatment protocol to clear them of infection^40^. All frogs survived in the sham exposed group while 12 individuals died following *B*. *dendrobatidis* exposure. These frogs, cleared of infection, were held for five months following confirmation of the absence of infection. All individuals were then euthanized with MS-222, weighed, dissected and the gonad mass was determined. The gonads were blot dried and weighed as indicated above.

All animals were held under a NSW scientific licence (approval number SL100421), and the experiments were conducted with the approval and in accordance of the University of Newcastle ACEC (approval number A-2008-165).

### Statistical analyses in Experiments 1 and 2

Infection load within the infected proportion of the population was analysed over time using a generalised linear mixed effects model (GLMM) in SAS (version 9.4) using the PROC MIXED procedure. Temperature, time and the interaction between the two were modelled as fixed effects with animal ID modelled as a random effect. Repeated measures over time were modelled with a residual covariance matrix with compound symmetry covariance structure. The infection load data were log transformed (molecules/μl + 0.1). Adding 0.1 to all infection load data allowed individuals that were negative for infection (i.e. 0 molecules/μl) to be log transformed.

The data for the proportion of individuals surviving across time in experiment 1 were analysed using a Log Rank test in a Kaplan-Meier survival analysis in SPSS (Version 21, IBM, New York). Post-Hoc pairwise comparisons of survival were made between the four treatment groups (+/− *B*. *dendrobatidis* exposure; 12 °C and 24 °C) using data for the proportion of individuals alive at 48 days.

The SMR data were analysed using a two-way analysis of variance (two-way ANOVA) in JMP (Version 11, SAS Institute, Cary, North Carolina) with temperature (12 °C or 24 °C), *B*. *dendrobatidis* exposure (infected or uninfected) and their interactions as the predictor variables. Individual body mass was taken into account by raising to the power of 0.75 (equation 1).

The fat mass data of frogs from experiment 1 were analysed using a two-way analysis of variance (two-way ANOVA) in JMP (Version 11, SAS Institute, Cary, North Carolina) where temperature (12 °C or 24 °C), *B*. *dendrobatidis* exposure (infected or uninfected) and their interactions were the selected predictor variables.

Ovary and testes mass data of frogs from experiment 2 were analysed separately using an analysis of covariance (ANCOVA) in JMP (Version 11, SAS Institute, Cary, North Carolina) where *B*. *dendrobatidis* exposure (infected or uninfected) was the predictor variable. The body mass of each individual was included as a covariate in order to account for size differences between frogs. The weight of the individual was regressed against the weight of the ovaries or testes and separated by *B*. *dendrobatidis* exposure using a simple linear regression.

## Results

### Experiment 1: Sublethal *B*. *dendrobatidis* infection of sexually immature *L*. *aurea*

**Figure 1:**
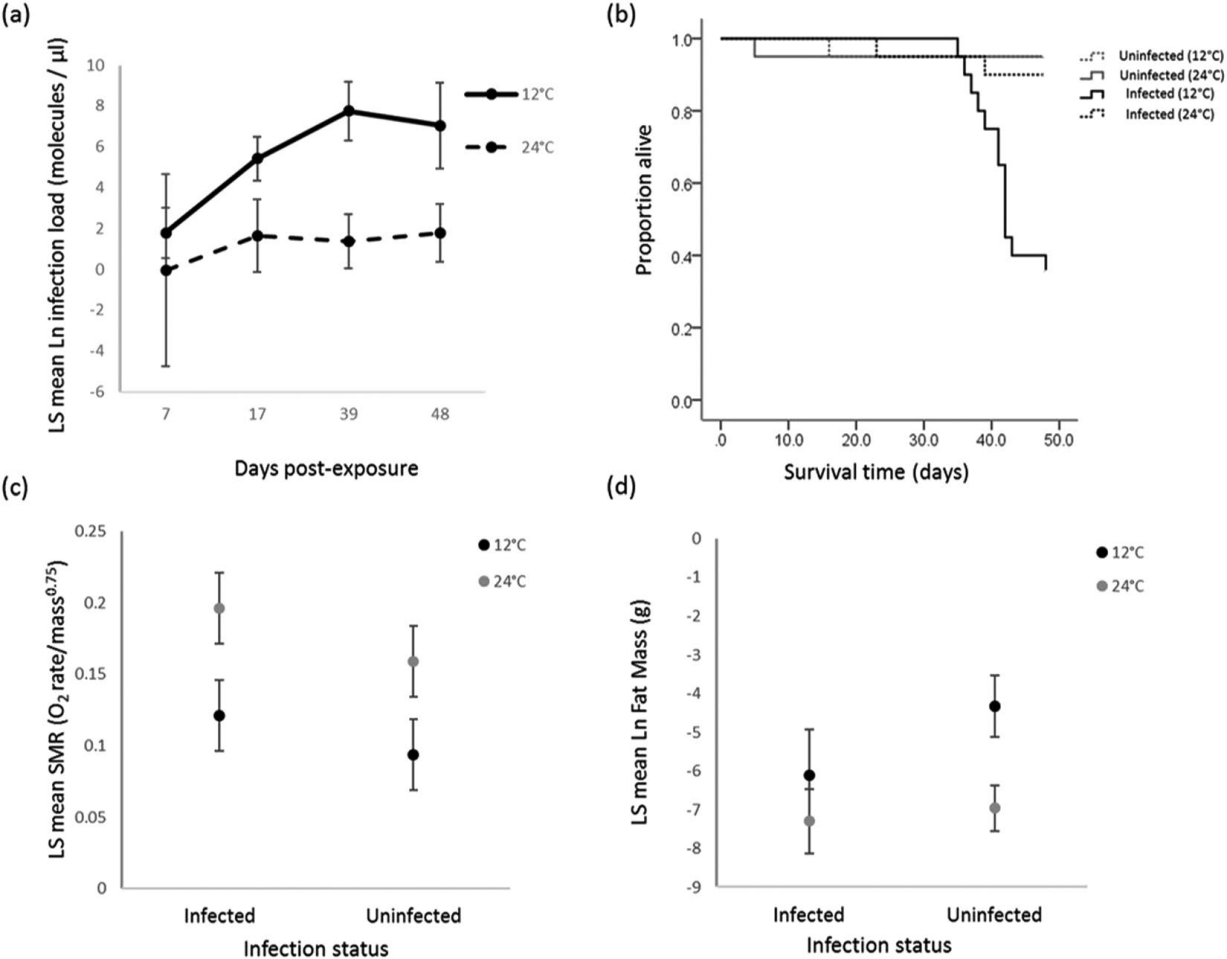
Effect of temperature and *Bd* infection in *L. aurea* for experiment 1. (**a**) Effect of temperature (12 °C or 24 °C) on mean infection load in *Bd* infected frogs. Values are least squares means ± 1CI (95%); (**b**) Effect of temperature (12 °C or 24 °C) and *Bd* infection (infected or uninfected) on survival over 48 days; (**c**) Mean SMR in infected and uninfected frogs at 12 °C or 24 °C. Values are least squares means ± 1CI (95%); (**d**) Mean fat mass in infected and uninfected frogs at 12 °C or 24 °C. Values are log transformed least squares means ± 1CI (95%).

All *B*. *dendrobatidis*-exposed frogs at 12 °C were infected (n = 20), while 16 of the 20 individuals held at 24 °C became infected for the duration of the study. These four uninfected frogs were removed from the analyses. All unexposed frogs tested negative for infection. Temperature significantly affected the growth of *B*. *dendrobatidis*; infection loads were much higher in infected frogs at 12 °C than 24 °C (F = 22.28, df = 1, *P* = 0.001; Fig. 1a). Days post exposure also significantly affected the growth rate of *B*. *dendrobatidis* with infection loads increasing over time (F = 2.86, df = 3, *P* = 0.045, Fig. 1a). Temperature also interacted with days post exposure; infection loads were greater and increased faster in frogs held at 12 °C compared to those held at 24 °C (F = 3.12, df = 3, *P* = 0.034, Fig. 1a). There was no significant difference between infection loads at 7 days compared to 48 days in frogs held at 24 °C (t-value = −0.44, df = 57.3, *P* = 0.659, Fig. 1a), whereas the infection loads between 7 and 48 days post-exposure significantly differed in frogs held at 12 °C (t-value = −4.97, df = 55.3, *P* < 0.001, Fig. 1a).

After 48 days, infected frogs that were held at 12 °C had significantly lower survivorship (30%), than uninfected frogs from both temperature treatments (95%), and frogs infected with *B*. *dendrobatidis* at 24 °C (90%) (χ^2^ = 31.77, df = 3, *P* < 0.001; Fig. 1b).

Infected individuals had a mean SMR of 0.16 O_2_ mL/h/g 0.75 ± 0.01 which was higher than uninfected individuals (0.13 O_2_ mL/h/g 0.75 ± 0.01) (F = 7.03, df = 1, *P* = 0.013, Fig. 1c). The SMR of individuals at 24 °C was 0.18 O_2_ mL/h/g 0.75 ± 0.01, which was significantly higher than those held at 12 °C (0.11 O_2_ mL/h/g 0.75 ± 0.01, F = 33.81, df = 1, *P* < 0.001). However, there was no interaction between temperature and infection status on SMR (F = 0.17, df = 1, *P* = 0.684, Fig. 1c).

Infected frogs had less stored fat (0.002 g ± 0.003) compared to uninfected individuals (0.01 g ± 0.002) (F = 5.19, df = 1, *P* < 0.03, Fig. 1d). Total fat mass was highly dependent on temperature; frogs maintained at 12 °C had far heavier fat bodies (0.02 g ± 0.003) than those kept at 24 °C (0.001 g ± 0.002) (F = 17.65, df = 1, *P* < 0.001, Fig. 1d). However, the interaction between temperature and infection status was non-significant (F = 3.32, df = 1, *P* = 0.078).

### Experiment 2: Long-term sublethal effects of a previous *B*. *dendrobatidis* infection in *L*. *aurea*

**Figure 2:**
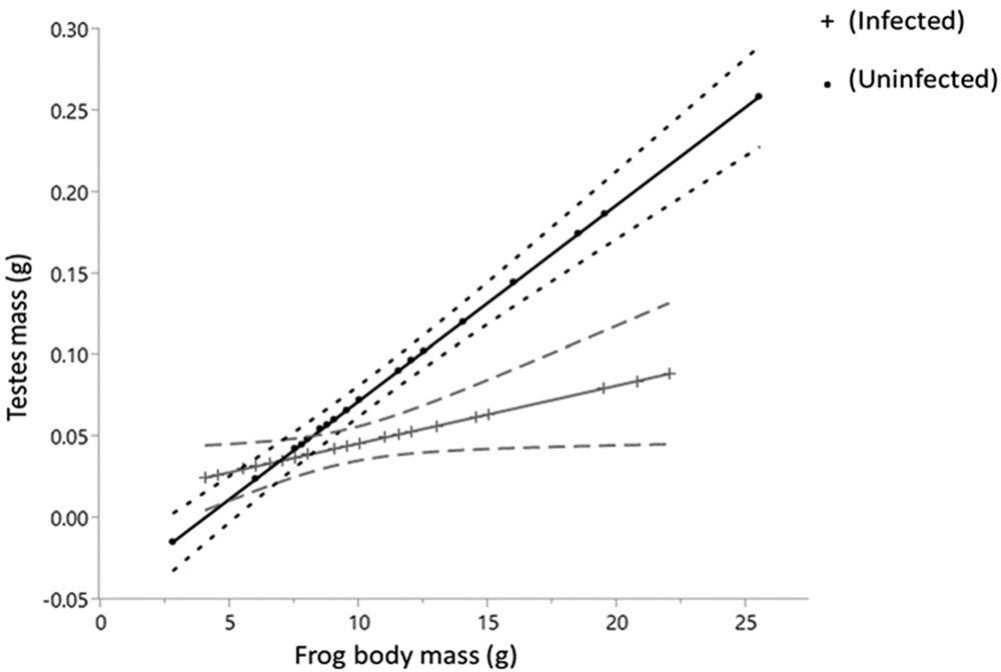
Regression of *L*. *aurea* testes mass data from experiment 2 against body weight. Broken lines = 95% confidence of fit.

Previously infected and non-infected male frogs had similar body weights (F = 0.60, df = 1, *P* = 0.552). Testes mass was positively correlated with the frog body mass (F = 70.60, df = 1, *P* < 0.001) and mean testes mass was significantly lower in the previously infected frogs (0.044 g ± 0.005) than uninfected frogs (0.078 g ± 0.005) (F = 14.35, df = 1, *P* = 0.001). The testes mass of previously infected frogs was much smaller for equivalent body weight than uninfected frogs causing an interaction between body mass and testes mass (F = 20.91, df = 1, *P* < 0.001; Fig. 2).

Female body weight was similar between the infected and uninfected groups (F = 0.36, df = 1, *P* = 0.552). Ovary mass positively correlated with the frog body weight (F = 93.57, df = 1, *P* = 0.001). However, there were no significant differences in ovary mass between infected and uninfected females (see Supplementary Fig. S3 online) (F = 3.71, df, = 1, *P* = 0.065).

## Discussion

*Litoria aurea* experienced a range of sublethal impacts during and following infection with *B*. *dendrobatidis*. Metabolic activity increased and abdominal fat bodies decreased in juvenile frogs during infection whereas previously infected males had smaller testes. These effects are likely to translate to fitness costs in wild populations. Temperature had direct effects on physiological processes (e.g. metabolic rate and fat bodies) but did not interact with infection load to change the magnitude of physiological effects. Together, these experiments demonstrate an energetic cost to sublethal *B*. *dendrobatidis* infection, which suggests a physiological trade-off occurs to infected individuals regardless of the outcome of infection.

Infection loads were highest in the coolest temperature treatment, despite the warmer temperature group occurring within the optimum temperature range for *B*. *dendrobatidis* growth in culture (24 °C)^18^. This may be a result of life-history trade-offs in the growth of *B*. *dendrobatidis* zoospores, which though encysting and developing into zoosporangium faster at 17–25 °C, produce more zoospores that remain infectious for a longer period at 7–10 °C^8^. An additional explanation for this counter-intuitive effect may be owing to a decreased immune system function of frogs in response to cold-exposure^20,41,42^, which could allow *B*. *dendrobatidis* to proliferate in the host. The effects of temperature were clear in the response variables we measured, where frogs that are normally metabolically unchallenged at 12 °C were affected by the higher infection loads. In contrast, frogs held at 24 °C have higher temperature-driven metabolic rates, but experienced much lower infection loads. The disproportionate, temperature-dependent growth of *B*. *dendrobatidis* highlights the importance of investigating responses of the host in the context of the ambient temperature, especially if the effects are to be extrapolated to wild populations. Furthermore, the increased SMR of infected frogs can have major implications on host fitness if frogs are concurrently infected during the higher Summer temperatures. It is unclear how transferable these findings are for other amphibian species, and individual species responses are likely to involve interactions between the range of thermal tolerance of species and populations and their immune response to *B*. *dendrobatidis* infection. For example, alpine species with low tolerances to high temperatures and a low capacity to tolerate infections are likely to respond very differently to *L*. *aurea*, which can self-cure infections under specific conditions^23,43^.

Elevated SMR and reduced abdominal fat in frogs with active *B*. *dendrobatidis* infections supported the hypothesis that infection has physiological effects even when the infection has not progressed to chytridiomycosis; this adds to the growing body of literature demonstrating sublethal impacts of *B*. *dendrobatidis*. In tadpoles, reduced developmental rates are probably driven by reduced activity and efficiency when foraging^44^, possibly owing, in part, to physical degradation of mouth parts. In frogs, the increase in standard metabolic rate contributes to an individual’s energy budget and represents a fixed cost of maintaining the body’s processes^45^ and the increase in infected individuals may reflect the body’s requirement to work harder through damage caused by infection. Initial host invasion of susceptible and tolerant hosts is superficially similar and involves varying levels of disruption to epidermal cells^46^, resulting in increased skin sloughing^47^, which may direct resources away from other core functions and thus increase the SMR. The increase in SMR is the likely mechanism behind the reduction in abdominal fat because infected individuals had higher metabolic rates and were therefore using more energy, which is acquired through ingested prey and is removed from accumulated body energy stores in the form of fat bodies. Alternatively, it is possible that infected individuals did not eat as much as uninfected frogs, as we would expect if their foraging efficiency was reduced, but we did not see evidence of this during our controlled experiment. Determining the relationship between high SMR and total fitness in infected individuals would allow us to better understand the consequence of this physiological effect.

Abdominal fat bodies are important for both gonadal development^48^ and the production of gametes^49^ and so it is plausible that *B*. *dendrobatidis* infection reduced the amount of available energy for reproductive growth and development. Gonads of males previously infected with *B*. *dendrobatidis* had smaller testes despite a five month recovery period following clearance of infection. It is possible that the reduced testes size was a result of the energetic impacts that reduced fat bodies and increased SMR in experiment 1. Small testes may have fitness consequences because testis mass correlates with hormone production and reproductive effort^50^. However, testes size is a crude measure of short-term changes in reproductive effort and it is possible that increased spermatogenesis still occurred^33^ but did so within smaller testes. Alternatively, increased spermatogenesis may only occur once chytridiomycosis has progressed as is reflected in the condition dependent vocalisation effort of *Litoria rhecoloa* when infected with *B*. *dendrobatidis*^31^. Our results do not reflect the terminal investment hypothesis suggested in other studies, e.g. increased testes width of infected *Rana pipiens*^28,33,51^, but support studies suggesting supressed reproduction occurs through down regulation of sex hormones^52^. The reduced growth rate of the testes could have major implications for the reproductive output of a host population. Further investigation of this phenomenon in *L*. *aurea* is required to determine whether smaller testes are associated with a lower sperm production; this is obviously an important measure of reproductive function to understand in the context of overall male reproductive fitness.

The reduced abdominal fat and testes mass in *B*. *dendrobatidis-*infected frogs shows support for an energetic tradeoff, favouring other systems over reproduction. While there is the possibility that these individuals may still have the capacity to reproduce physiologically, the reduced energy supplies may affect their immediate survival^53^ and the ability to carry out expensive reproductive behaviours such as vocal calling and mate-seeking^50^. Such a reduction in fitness of individuals could possibly result in reduced breeding and increased mortality from secondary causes (i.e. predation), which may then lead to less stable populations. Host-populations that are then able to persist with low infection loads may still be at risk of decline despite the absence of mortality events attributed directly to *B*. *dendrobatidis* infection. Further research is needed to understand whether the fitness consequences of *B*. *dendrobatidis* infection on the individual demonstrated in this study are transferrable to wild populations of *L*. *aurea* and directly impact fitness at the population level.

The fact that females infected with *B*. *dendrobatidis* had similar ovary size than uninfected females, despite the differences observed in the male gonad mass, is interesting. Our results differ from those of infected *Litoria verreauxii alpina*, which had larger ovaries and oviducts in infected animals suggesting terminal investment occurs in diseased individuals^28^ in this species. We are unsure why such sex differences occurred in this species, but it is an area worthy of future investigation. There has been little research in this area to date and further studies should aim to resolve mechanisms and effects on female reproductive biology in other species.

In conclusion, our findings reveal further insights into the sublethal effects of *B*. *dendrobatidis* infection on both host physiology and reproductive potential. The colonisation of the amphibian chytrid fungus and subsequent battle between host and parasite have clear costs to *L*. *aurea*, regardless of infection outcome and there is mounting evidence that frogs do not have to be susceptible to chytridiomycosis to be at least temporarily affected by the fungus^28,54,55^. This is an important consideration in our understanding of the amphibian chytrid fungus, especially where declines have occurred without an obvious mortality of, or disease risk to, the host. Physiological effects involving increased SMR, reduced abdominal fat and reduced testes mass seem likely to translate to consequences for wild populations in terms of reduced total fitness, but this relationship is often assumed but rarely confirmed. Ecological studies that can incorporate infection dynamics into host fitness outcomes are necessary to provide a comprehensive understanding of the magnitude of these physiological effects.

## Electronic supplementary material

Figure 3

## Data Availability

We intend for the data of this study to be archived at the Dryad Digital Repository.
