# Supplementary material for: Interaction between temperature and sublethal infection with the amphibian chytrid fungus impacts a susceptible frog species
Source: Sci Rep. 2019 Jan 14;9:83. doi: 10.1038/s41598-018-35874-7 (PMC6331562; doi:10.1038/s41598-018-35874-7)
Supplement: Supplementary file 1 — Figure 3 [file 41598_2018_35874_MOESM1_ESM.pdf]

Supplementary information

Title: Interaction between temperature and sublethal infection with the amphibian chytrid fungus impacts a susceptible frog species.

Authors: Lachlan Campbell[1*], Deborah S. Bower[1, 2], Simon Clulow[1,3], Michelle Stockwell[1], John Clulow[1], Michael Mahony[1]

Corresponding author: LCampbell@uon.edu.au

[1] School of Environmental and Life Sciences, University of Newcastle, Callaghan, Newcastle, 2300, NSW, Australia

[2]James Cook University, Townsville, 4811, Qld, Australia

[3] Department of Biological Sciences, Macquarie University, Sydney, NSW, 2109 Australia

Figure and caption

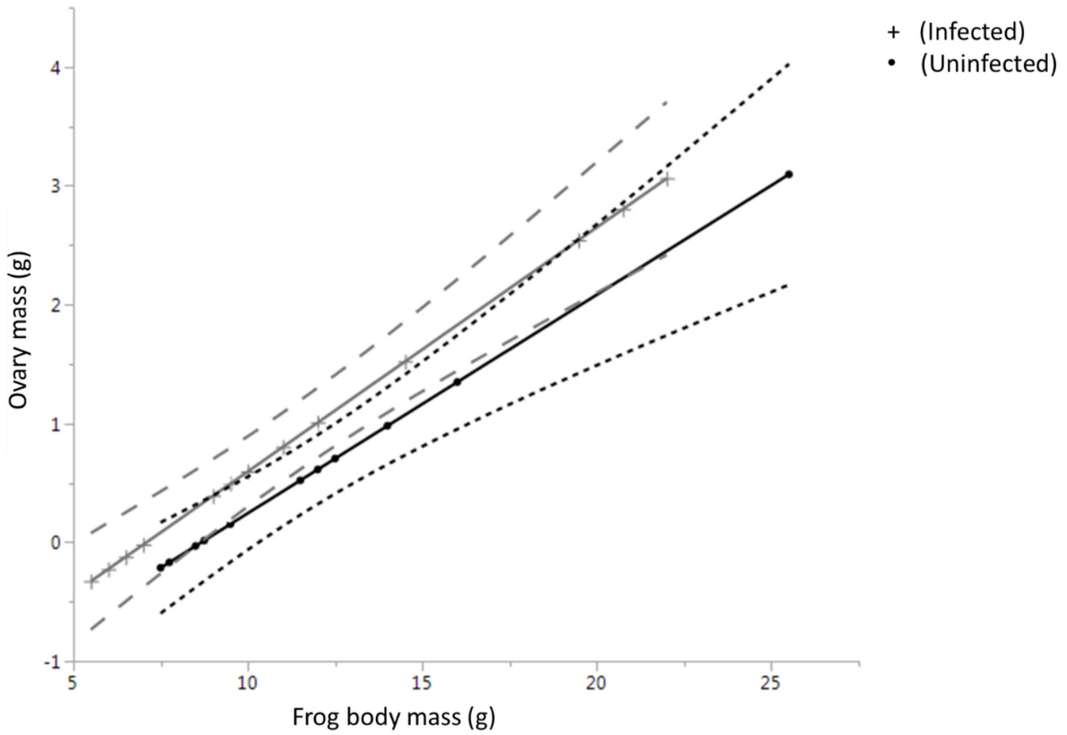

Figure 3: Regression of *L. aurea* ovary mass data from experiment 2 against body weight. Broken lines = 95% confidence of fit.
